# Artificial Intelligence Assisting the Early Detection of Active Pulmonary Tuberculosis From Chest X-Rays: A Population-Based Study

**DOI:** 10.3389/fmolb.2022.874475

**Published:** 2022-04-08

**Authors:** Mayidili Nijiati, Jie Ma, Chuling Hu, Abudouresuli Tuersun, Abudoukeyoumujiang Abulizi, Abudoureyimu Kelimu, Dongyu Zhang, Guanbin Li, Xiaoguang Zou

**Affiliations:** ^1^ Department of Radiology, The First People’s Hospital of Kashi Prefecture, Kashi, China; ^2^ School of Computer Science and Engineering, Sun Yat-sen University, Guangzhou, China; ^3^ Department of Colorectal Surgery, The Sixth Affiliated Hospital, Sun Yat-sen University, Guangzhou, China; ^4^ Department of Radiology, Kashi Area Tuberculosis Control Center, Kashi, China; ^5^ Clinical Medical Research Center, The First People’s Hospital of Kashi Prefecture, Kashi, China

**Keywords:** tuberculosis, chest radiograph, machine learning, artificial intelligence, deep convolutional neural network

## Abstract

As a major infectious disease, *tuberculosis* (TB) still poses a threat to people’s health in China. As a triage test for TB, reading chest radiography with traditional approach ends up with high inter-radiologist and intra-radiologist variability, moderate specificity and a waste of time and medical resources. Thus, this study established a deep convolutional neural network (DCNN) based artificial intelligence (AI) algorithm, aiming at diagnosing TB on posteroanterior chest X-ray photographs in an effective and accurate way. Altogether, 5,000 patients with TB and 4,628 patients without TB were included in the study, totaling to 9,628 chest X-ray photographs analyzed. Splitting the radiographs into a training set (80.4%) and a testing set (19.6%), three different DCNN algorithms, including ResNet, VGG, and AlexNet, were trained to classify the chest radiographs as images of pulmonary TB or without TB. Both the diagnostic accuracy and the area under the receiver operating characteristic curve were used to evaluate the performance of the three AI diagnosis models. Reaching an accuracy of 96.73% and marking the precise TB regions on the radiographs, ResNet algorithm-based AI outperformed the rest models and showed excellent diagnostic ability in different clinical subgroups in the stratification analysis. In summary, the ResNet algorithm-based AI diagnosis system provided accurate TB diagnosis, which could have broad prospects in clinical application for TB diagnosis, especially in poor regions with high TB incidence.

## Introduction

Causing by *Mycobacterium tuberculosis* infection, pulmonary *tuberculosis* (TB) is a kind of dangerous airborne chronic respiratory infectious disease (Ragonnet et al., 2019). TB still remains a major problem of disease control and poses a threat to the health of the public in China (Tusun et al., 2021). The incidence of TB in Kashi was 250.4/100,000 in 2020, reaching 4.3 times the national average incidence. TB epidemic remains severe, especially in 12 counties/cities of Kashi prefecture, Xinjiang Uygur Autonomous Region (Tusun et al., 2021).

A triage test using chest radiography is utilized for patients with typical symptoms for TB or TB-related risk factors (Qin et al., 2021a). Both the shortage of experienced radiologist and the high inter-radiologist and intra-radiologist variability have been affecting the performance and generalizability of the chest radiography, especially in places with a high incidence of TB and without access to high quality medical service (Organization, 2016). However, during the last 10 years, the artificial intelligence (AI) aided diagnostics systems have been developing and evolving at an unprecedented pace, leading to its deployment and usage in clinical settings, and many medical image analyzing AI algorithms, which were based on deep learning and deep convolutional neural networks (DCNNs), were being utilized for radiographs reading at the same time (Rajpurkar et al., 2020; Qin et al., 2021b). Such deep learning and DCNN algorithms are able to distinguish the features and characteristics of the TB-related abnormalities in the chest X -ray photographs. Considering the great improvement of AI-assisted TB diagnosis, computer aided TB screening software is a better substitute for physicians in digital chest radiographs reading and analyzing, which was recommended by the updated guidelines of the World Health Organization (WHO) in March 2021 (Organization, 2021). However, there remains uncertain about what kind of algorithm or AI should be developed and put into clinical practice, since the WHO didn’t give detailed recommendations for specific products (Qin et al., 2021a).

To date, the majority of AI algorithms for TB diagnosis have been based on small groups of individuals. Considering that a large sample for training would further improve the performance of the AI algorithm, the deep convolutional neural network (DCNN), a kind of deep learning approach, has been widely utilized for analyzing medical images. Thus, this study explored the TB diagnosis ability of three kinds of DCNNs (Resnet, VGG, and AlexNet algorithms) based on chest X-rays of 10,000 individuals.

## Materials and Methods

### Study Setting and Population

**FIGURE 1:**
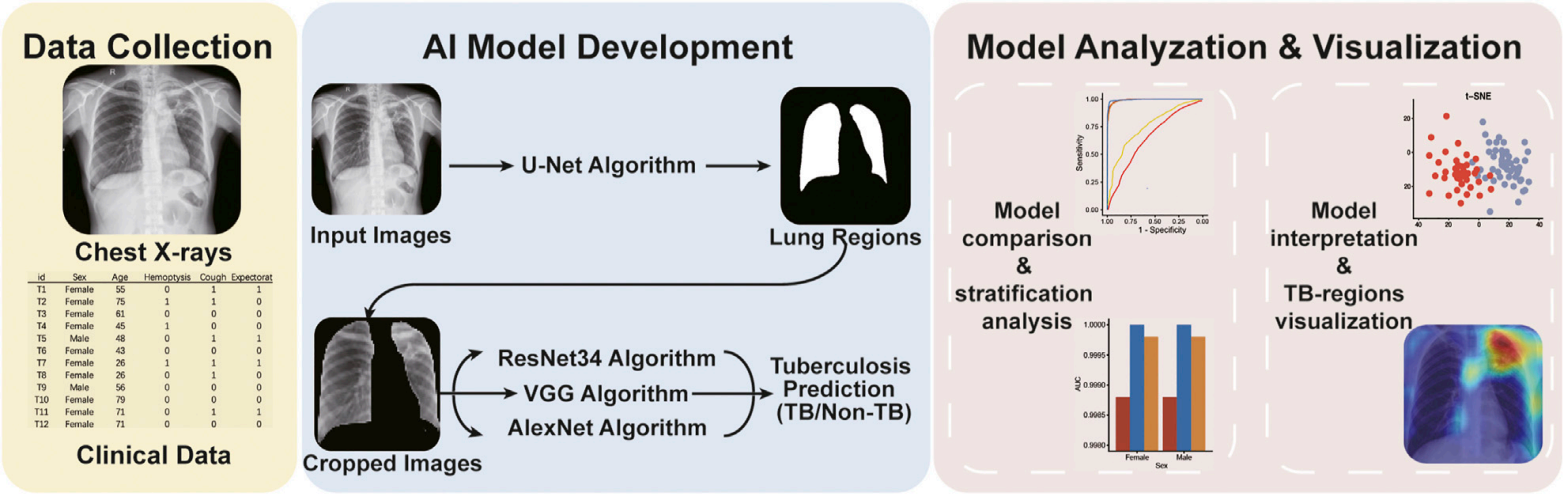
The workflow of the study.

In this retrospective study, we trained convolutional neural network-based AI algorithms to read chest X-rays for pulmonary TB diagnosis. The workflow of the study was showed in Figure 1. In total, 9628 X-ray images and corresponding clinical information were collected from individuals with and without TB in Kashgar, Xinjiang, China, between 2019 and 2020 (Table 1). The included cases were aged ≥15 years and underwent X-ray analysis. TB cases were diagnosed by experienced physicians based on the symptoms and the results of multiple tests and radiological examinations, including sputum culture or smear tests, Xpert tests, chest X-ray films, interferon gamma release assays and tuberculin skin tests and so on. In total, 5,000 images of TB cases and 4,628 images of non-TB cases were collected with privacy information removed and split into the training (*n* = 7,703) and testing sets (*n* = 1925) (Table 1).

**TABLE 1:** A summary of clinical characteristics of training and testing sets.

	Training set		Testing set	
	TB cases	Non-TB cases	TB cases	Non-TB cases
Sex				
Female	2,118	1885	463	478
Male	1882	1818	537	447
Age				
<65 years	2,297	1971	461	580
≥65 years	1703	1732	539	345
Symptoms				
Cough	3,304	6	782	8
Expectoration	2,726	4	606	1
Hemoptysis	461	5	128	4
Fever	1,563	4	429	0
Fatigue	1,143	0	248	0
Night Sweating	789	0	135	0
Bacteriological Test				
Sputum Culture/Smear Positive	807	0	297	0
Bacteriological Test Positive	1788	0	509	0
Bacteriological Test Negative	2,212	3,703	491	925
Total	4,000	3,703	1,000	925

Sputum Culture/Smear Positive: sputum culture positive or smear positive. Bacteriological Test Positive: sputum culture/smear positive or Xpert test positve. Bacteriological Test Negative: sputum culture negative, sputum smear negative and Xpert test negative.

### X-Rays Images Preprocessing

**FIGURE 2:**
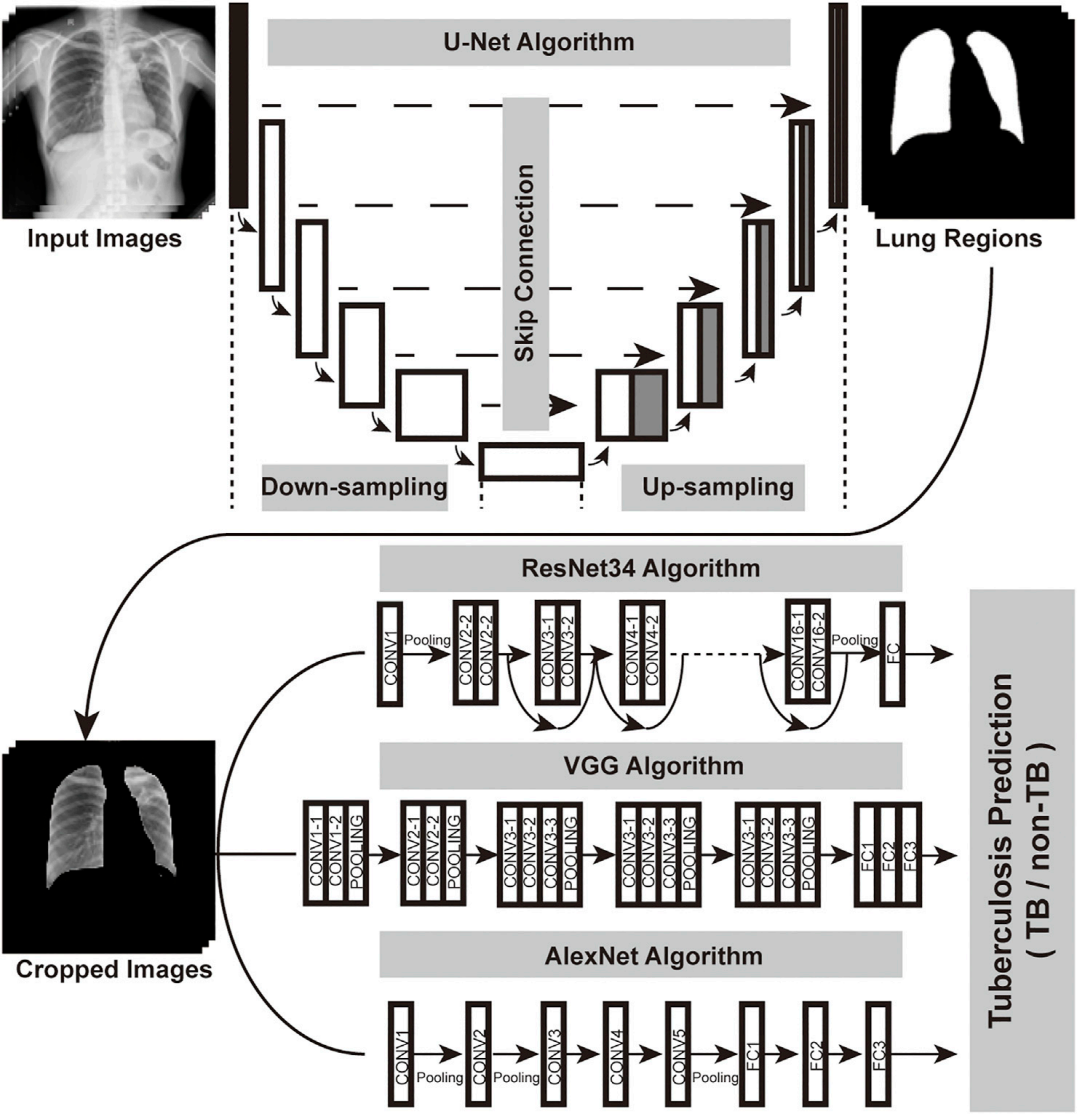
Overall structure of the DCNN-based AI diagnosis system. The workflow of the system could be divided into two parts: image segmentation network (U-Net), image classification network (ResNet or VGG or AlexNet). Regions of the lung in the original chest X-ray photographs were recognized by the U-Net. Then, the cropped and resized lung region images served as an input for image classification algorithms, which generated diagnoses.

As a tool widely used in medical image semantic segmentation, U-Net has been applied to extract semantic information and generate segmentation results (Ronneberger et al., 2015; Zunair and Ben Hamza, 2021). In order to focus on pulmonary TB-affected regions that appeared inside the lungs, U-Net was used for the lung segment before TB classification. After image cropping and resizing, the lung region images served as an input for the classification convolutional neural network (Figure 2).

### Development of AI Algorithms

For the classification network, we used ResNet34 (He et al., 2016), VGG (Simonyan and Zisserman, 2014) and AlexNet (Krizhevsky et al., 2012). The structures of the three convolutional neural networks were illustrated in Figure 2. We loaded the pretrained model in ImageNet and replaced the last linear classification layer with a new linear layer for negative (individuals without TB) or positive (with TB) prediction. The ResNet model was trained for 120 epochs in total with chest X-ray images of the training set (Supplementary Figure S1), setting the initial learning rate at 1e−3 and using the inverse learning rate decay schedule. VGG and AlexNet were also trained with the same settings, and then three AI algorithms were validated on the testing set.

### Comparison of AI Algorithms and Stratification Analysis

To evaluate the performance of the three models, the accuracy, sensitivity and specificity of the three models were calculated in both sets for comparison (Table 2), and the plots of receiver operating characteristic (ROC) curves with area under the curve (AUC) values were also generated with the pROC package. The AI algorithm with the best performance was chosen for the following analysis and visualization. To further investigate the reliability and robustness of the AI models, cases of the testing set were stratified into multiple subgroups based on their age, sex and respiratory symptoms, and then the AUC value of each model were calculated and compared within each subgroup with the pROC package.

**TABLE 2:** The performance of the models.

	Training set			AUC (95% CI)	Testing set			AUC (95% CI)
	Accuracy (%)	Sensitivity (%)	Specificity (%)		Accuracy (%)	Sensitivity (%)	Specificity (%)	
AlexNet	98.34	98.58	98.08	0.9988 (0.9984–0.9992)	95.06	93.20	97.08	0.9917 (0.9889–0.9945)
VGG	99.03	99.75	98.24	0.9998 (0.9997–0.9999)	94.96	94.20	95.78	0.9902 (0.9872–0.9932)
ResNet	99.92	99.90	99.95	1 (1–1)	96.73	95.50	98.05	0.9944 (0.9921–0.9967)

AUC, the area under the receiver operator characteristic curve; 95% CI: 95% confidence interval.

### Interpretability Analysis and Feature Visualization

After the comparison, ResNet algorithm was chosen for further interpretability analysis and feature visualization. After the global average pooling layer of ResNet, we obtained a 512-dimensional vector for each image. To visualize the learned feature, we used the t-SNE method, which could reduce the high-dimensional vector to a low-dimensional vector (Van et al., 2008). Here, we reduced each vector to 2-dimension and therefore, visualized the differences in high-dimensional complex features between the positive and negative images captured by the AI algorithm. We also obtained sets of feature maps from the last convolution layer of ResNet. Then, class activation maps (CAMs) were generated by the linear combination of the fully connected layer weights and feature maps (Zhou et al., 2016). Discriminative regions of the CAMs were in red (hot areas), indicating the AI-predicted TB regions.

## Results

**FIGURE 3:**
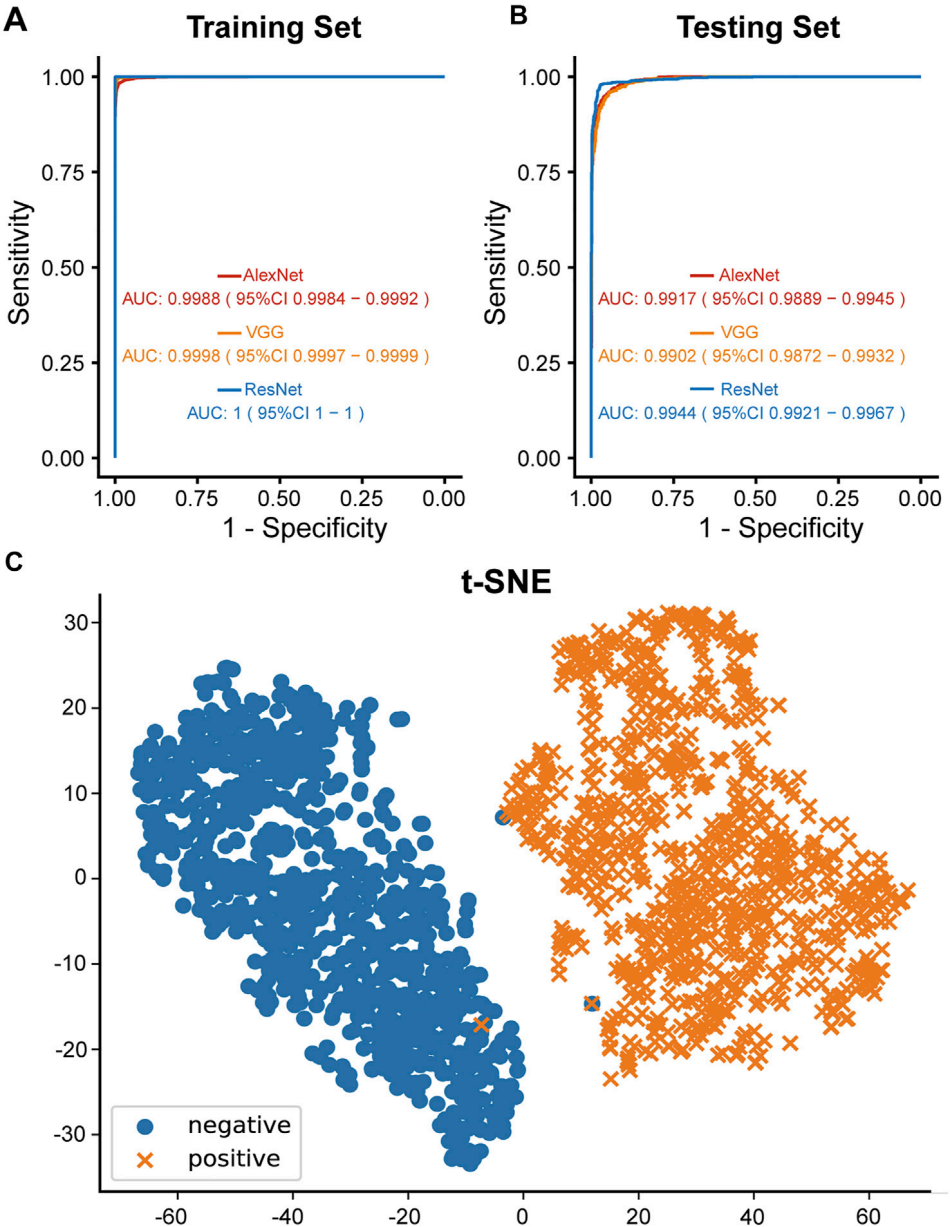
Diagnostic ability of the AI models. **(A)** ROC curves of three different AI models of the training set. **(B)** ROC curves of three different AI models of the testing set. **(C)** Diagnostic ability of ResNet algorithm visualized by t-SNE algorithm. Blue and orange dots indicated TB and non-TB cases of the testing set, respectively.

In the beginning of the study, 9628 X-ray images with detailed clinical information were collected (Figure 1). Clinical characteristics of the collected cases were summarized in Table 1. Three different AI algorithms were trained on a large dataset containing 4,000 images from patients with TB and 3,703 images from individuals without TB (Figure 2). After training, these AI algorithms were compared to each other on the testing set and the training set. The accuracy, sensitivity and specificity of the three algorithms were all higher than 94% when they were tested on the testing set (Table 2). The ResNet model had the strongest diagnostic ability among the three AI algorithms, whose accuracy, sensitivity and specificity reached 96.73, 95.50 and 98.05%, respectively. The performance of the AI models were further investigated with ROC curves. All the AI algorithms performed well in both training set and testing set (Figures 3A,B), with AUC values higher than 0.99 (Table 2). The AUC value of the AlexNet, VGG and ResNet reached 0.9917, 0.9902 and 0.9944, respectively on the testing set. These results suggested that the ResNet algorithm outperformed the rest models, demonstrating its excellent diagnostic value for TB and was selected for further analysis and visualization.

To understand how the AI algorithms distinguished TB radiographs from non-TB ones, the dimensional reduction t-SNE algorithm was applied to reduce the high-dimensional differences in visual-semantic information aggregated by the AI algorithm between TB-positive and TB-negative images into a two-dimensional plot. Here, taking ResNet as an example, an evident boundary was observed between the positive and negative image groups, while clustered distributions within each group were also observed, indicating that the ResNet algorithm succeeded in recognizing shared features of TB-positive images and features that distinguished the positive and negative images (Figure 3C).

**FIGURE 4:**
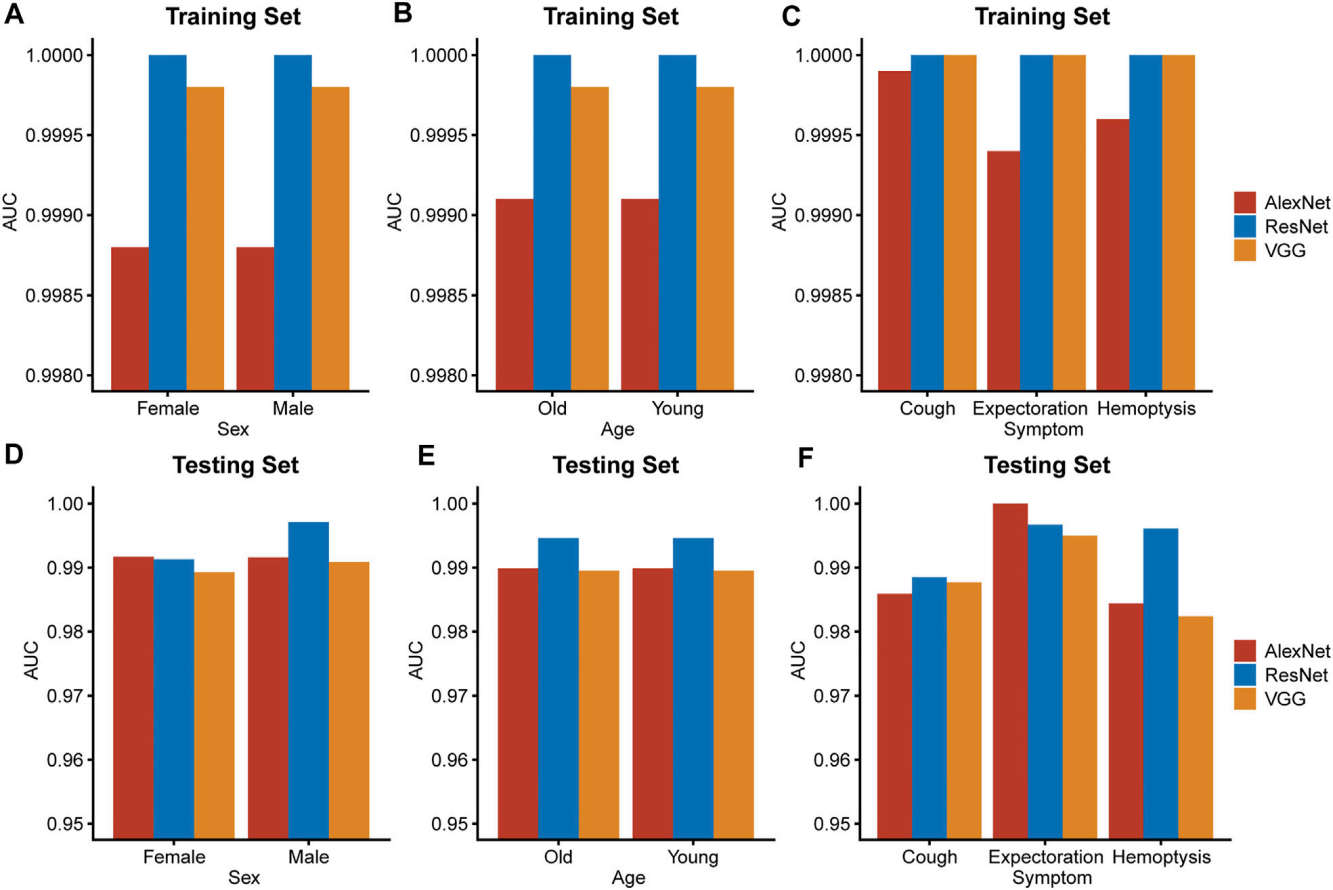
Stratification analysis. Subgrouping by important clinical characteristics, including sex **(A,D)**, age **(B,E)** and respiratory symptoms **(C,F)**, AUC values of the three models were calculated and compared in both sets. Young: under 65 years old. Old: 65 years old or over 65 years old.

Apart from evaluating the diagnostic accuracy on the entire testing set or training set, stratification analysis was also conducted to verified the reliability and the robustness of the ResNet algorithm. The ResNet model was the best performing model with highest AUC value in all subgroups of the training set and most subgroups of the testing set (Figure 4). The result of the stratification analysis suggested that the ResNet algorithm was capable of providing accurate TB diagnoses for people of all ages and both sexes or patients with different respiratory symptoms.

**FIGURE 5:**
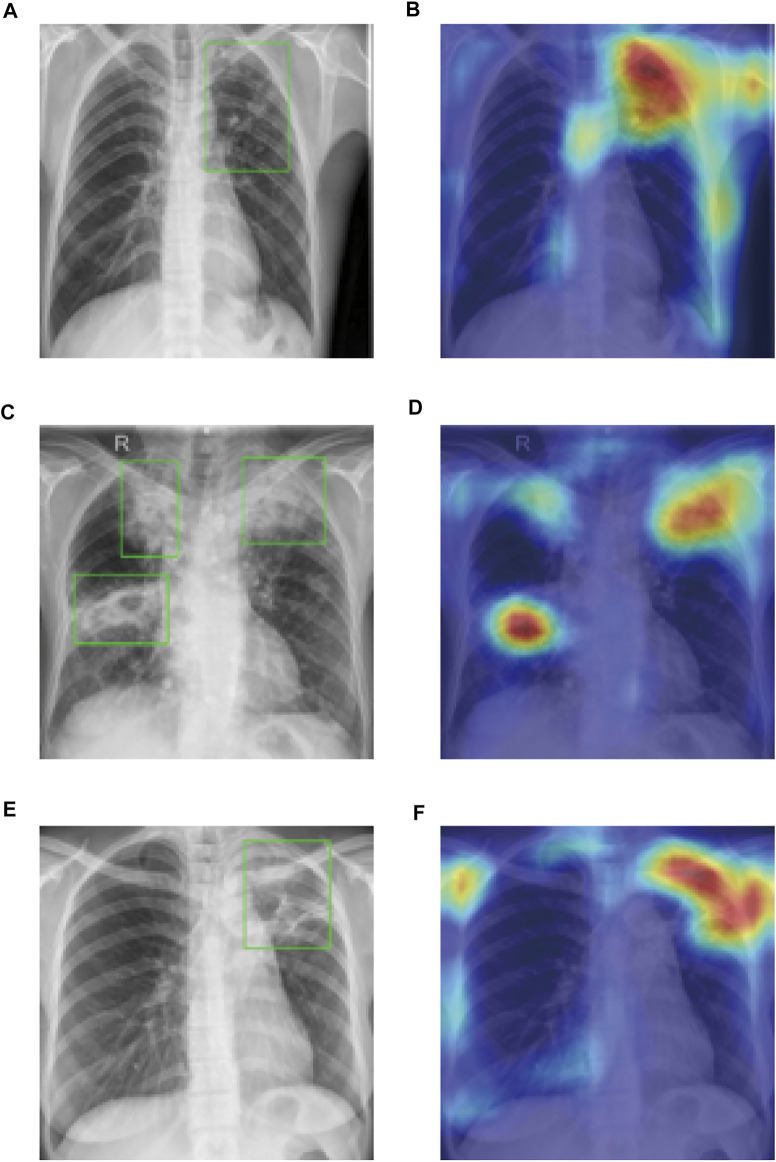
CAMs generated by ResNet matched the precise regions of TB abnormalities. Bounding boxes [in **(A,C,E)**] meant the regions of abnormalities identified by doctors and hot regions [in **(B,D,F)**] showed the discriminative regions generated by AI algorithm.

In order to step further in assisting the TB diagnosis, we visualized the ResNet algorithm recognized TB affected regions of the chest radiographs using the OpenCV package. Masked with red and alpha-blended with the black-and-white input X-ray image, “hot regions” drawn by AI provided accurate disease-affected areas and indicated high consistency with the TB regions mapped by experienced physicians and radiologists with bounding boxes (Figure 5). Our method not only provided a correct diagnosis of pulmonary TB but also identified precise TB regions with a heatmap, which has great potential in assisting the diagnosis of TB as an interpretable and reliable AI algorithm.

## Discussion

As an effective method for TB screening and diagnosis, chest radiography is recommended by multiple clinical guidelines despite its high inter-radiologist and intra-radiologist variability, moderate specificity and other limitations. In contrast to manual diagnosis, deep learning based computer-assisted diagnostic systems have the potential to overcome the aforementioned drawbacks and provide professional diagnosis for TB. Here, we established a ResNet-based chest X-ray AI diagnosis system for TB, which provided accurate diagnoses and was capable of serving as triage tests at the bedside.

A previous study has reported that the AlexNet based machine learning algorithm can accurately classify TB based on chest radiography (Lakhani and Sundaram, 2017). Here, our results indicated that the ResNet-based AI was superior to AlexNet and VGG, which suggested that the ResNet diagnosis system would better assist physicians in diagnosing TB.

Low interpretability is one of the major inherent problems of the machine learning models, including deep learning algorithms. Due to the complex calculating process and tremendous amount parameters of the neural networks, it remains difficult for us to learn about how they work and why they come to certain conclusions that remarkably similar to human experts’ opinions, suggesting that the neural networks, especially deep neural networks, are so-called black boxes (Wang et al., 2021). Even though depth of the DCNNs are becoming sheerer and sheer, many tools, including t-SNE and CAMs, are still available for the visualization of neural networks and breaking up the black boxes, convincing us that the neural network algorithm have the ability to recognized features of abnormality in the medical images rather than nonrelevant parts of the graphs. In this study, we wanted to know whether the trained DCNN based AI algorithm was focusing at regions of TB-associated abnormality in the lung. As illustrated in Figure 5, the discriminative regions, which were recognized by AI and masked with red, were the exact TB regions recognized and identified by doctors. This indicates that the ResNet-based AI algorithm not only provides doctors with highly accurate diagnoses but also interpretable marks of TB regions, which is of great help in analyzing chest X-ray images and recognizing TB in patients.

As a relatively remote and poor region with scarce medical resources in the past years, Xinjiang has been troubled by the continuous spread of TB (Zheng et al., 2021). Despite of financial difficulties, Xinjiang has managed to carry out many new policies and plans to boost investment in TB prevention and control, aiming at the early detection and proper treatment of TB cases. Early diagnosis of active TB is the key to controlling the rapid rise of TB incidence. Considering the excellent performance of the ResNet-based AI diagnosis system, it would greatly prompt the early diagnosis of active TB and help in preventing the spread of TB in Xinjiang.

However, there are several limitations in the study. First, although sputum culture is the gold standard for active pulmonary TB diagnosis, some patients of pulmonary TB have negative sputum culture results. Taking the results of multiple tests and typical clinical manifestation together, patients of TB were diagnosed, which remains risks of misdiagnoses and producing wrong labels for the chest radiographs. Besides, the chest radiographs were collected in two hospitals, suggesting that differences might exist between images captured in the two centers. In addition, we limited the study population to people aged ≥15 years, which also limited the generalizability of our AI diagnosis system towards pediatric cases.

In conclusion, our study established a ResNet-based AI diagnosis system that was effective in diagnosing active TB from chest radiographs without external clinical information assistance.

## Data Availability

The clinical data and chest radiographs are not publicly available for patient privacy protection purposes. Any individual affiliated with an academic institution may request access to the original images and clinical data from the corresponding author (MN) for non-commercial, research purposes.

## Abbreviations

AI, artificial intelligence; AUC, area under the curve; CAMs, class activation maps; DCNN, deep convolutional neural network; ROC, receiver operating characteristic; TB, *tuberculosis.*
