# Supplementary material for: Artificial Intelligence Assisting the Early Detection of Active Pulmonary Tuberculosis From Chest X-Rays: A Population-Based Study
Source: Front Mol Biosci. 2022 Apr 8;9:874475. doi: 10.3389/fmolb.2022.874475 (PMC9023793; doi:10.3389/fmolb.2022.874475)
Supplement: Supplementary file 1 [file DataSheet1.doc]

Supplementary Material


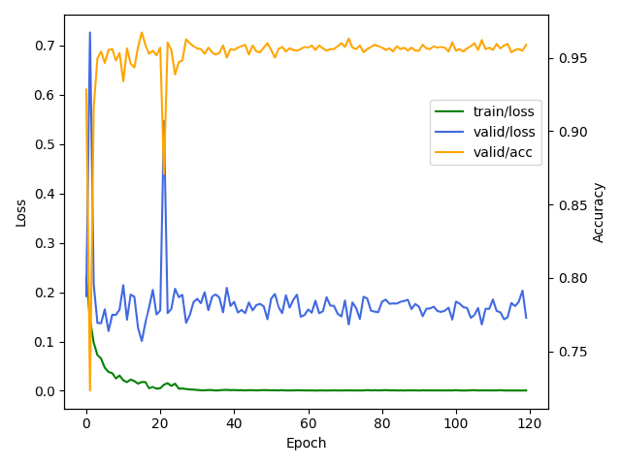


**Supplementary Figure 1.** The training loss and validation loss values after some runs are shown.
